# Microbes affected the TYLCCNV transmission rate by the Q biotype whitefly under high O_3_

**DOI:** 10.1038/s41598-017-14023-6

**Published:** 2017-10-31

**Authors:** Yanyun Hong, Tuyong Yi, Xiaoling Tan, Jianwei Su, Feng Ge

**Affiliations:** 1 Hunan Provincial Key Laboratory for Biology and Control of Plant Pests, College of Plant Protection, Hunan Agricultural University, Changsha, China; 2 State Key Laboratory of Integrated Management of Pest Insects and Rodents, Institute of Zoology, Chinese Academy of Sciences, Beijing, China

## Abstract

Ozone (O_3_) is a major air pollutant that has a profound effect on whole ecosystems. In this study we studied how hO3 affected the transmission of the *Tomato yellow leaf curl China virus* (*TYLCCNV*), a begomovirus, by the Q biotype *Bemisia tabaci* in a persistent, circulative manner. We found hO3 affected the transmission of *TYLCCNV* via the effect of it on the microbial community of the transmitting insect, such as *Candidatus Hamiltonella*, *Ralstonia*, *Diaphorobacter*, *Caldilineaceae*, *Deinococcus*, *Rickettsia*, *Thysanophora penicillioides* and *Wallemia ichthyophaga*. We concluded that hO_3_ decreased the resistance of acquiring virus tomatoes, and decreased the immune response and increased the endurance to extreme environments of viruliferous whiteflies by altering the composition and abundance of the microbial environments inside the body and on the surface of whitefly, as a result, it enhanced the TYLCV transmission rate by the Q biotype whitefly.

## Introduction

The global atmospheric concentration of ozone (O_3_) has risen from less than 10 ppb a century ago to 40–60 ppb at present, and it continues to increase at an annual rate of 1~2%^1^. In fact, in the forested regions of North America, the concentration of O3 reached 200 ppb in 1982^2^, and the maximum atmospheric concentration in Beijing was 273–477 ppb in July 2000^3,4^. O3, as a major air pollutant, affects some biological life on some organisms^5^. Ogawa *et al*. and Cui *et al*. found that O3-induced plants accumulated SA^6,7^. Salicylic acid (SA) plays a central role in the plant disease-resistance response, including the resistance against a broad spectrum of pathogens^8^. Deng *et al*. found that SA induced SAR to resist the tobacco mosaic virus (TMV)^9^. Sade *et al*. showed that SA was involved in tomato resistance to TYLCV^8^. Whether high O3 altered the transmission rate of plant virus by accumulating SA is unclear.

Ozone not only affects plant SA resistance, but also affects the composition and abundance of microbiology in the body and on the surface of insects^10^. And symbiotic microorganisms that act as an essential part of an insect’s structure and function have profound effects on the host insect’s biology^11–13^. When these microorganisms are removed or their composition is dramatically altered, the functions of the host insects demonstrate dysbiosis^14^. For example, germ-free Drosophila displayed delayed development, altered nutrient allocation and metabolic rates, and depressed gut immunity^15–17^. Some types of endosymbionts may regulate plant defenses. For example, the tomato psyllid, Bactericera cockerelli, reduced the expression of the tomato defensive pathway gene via the bacterial endosymbiont ‘*Candidatus Liberibacter psyllaurous*’^18^. Such changes maybe indirectly influence the transmission rate of a virus^19^. Moreover, endosymbionts can affect the transmission of a virus by means of direct participation. For example, Gottlieb *et al*. found that the endosymbiotic bacteria *Hamiltonella* from B biotype *Bemisia tabaci* in Israel produced the GroEL protein, which interacted with the TYLCV coat protein, thus enhancing the transmission efficiency of TYLCV^20^. Several studies have proved that endosymbiotic bacteria *Rickettsia* and *Hamiltonella* took part in TYLCCNV transmission via the Q biotype whitefly in China^21–24^. Zhu *et al*. demonstrated that a number of virulence-related genes were observed in the *Rickettsia* genome from *Bemisia tabaci* in Israel and China^25^. Moreover, fungi can directly assist the host in overcoming plant resistance; some fungi are associated with insects that can alter nitrogen directly from the insects to the plants and receive carbon (carbohydrates), in return, which can affect plant SA resistance, and some fungi can produce antibiotics that could affect a host insect’s immunity, which can indirectly affect the effectiveness of virus transmission^26–28^. Andrew & Lilleskov found that elevated CO2 (750 PPb) and O3 (80 PPb) affected the fungi community composition and sporocarp productivity^29^. Our previous study proved that high O3 (280 ± 20 PPb) significantly altered the abundance of microbes (bacteria and fungi) in the body and on the surface of the Q biotype whitefly^10^. Whether O3, as an environmental stress, alters the abundance and composition of the microbes associated with insects, and as a result, affects the transmission rate of plant virus by altering plant resistance, insect immunity, is unclear.

*Tomato yellow leaf curl virus* (TYLCV), a devastating plant virus, has caused hundreds of millions of dollars of crop damage in America^30^. TYLCV includes *Tomato yellow leaf curl Sardinia virus* (TYLCSV), *Tomato yellow leaf curl virus* (TYLCV) and *Tomato yellow leaf curl China virus* (TYLCCNV), which were first identified in Italy, Israel and China, respectively^31^. TYLCCNV is predominantly found in China and is specifically transmitted by the Q biotype whitefly. Several studies provided that the Toll-like signaling of whitefly affected the TYLCV transmission rate^23,32,33^. Whether and how high O_3_ affect the transmission rate of TYLLCV is unclear.

To highlight the main effects of ozone and to exclude other environmental factors, we set our O_3_ treatment concentration at 280 ± 20 ppb (hO_3_). In this study, our hypothesis was that hO3 would alter TYLCCNV transmission by the Q biotype *B*. *tabaci* via affecting the resistance of the receptor plants, the changes in immunity and the microbial communities of the insect vectors. To test this hypothesis, we determined the effects of hO_3_ on the following characteristics of the Q biotype *B*. *tabaci* and tomato: (1) comparison of the TYLCCNV content between high O_3_ and control O_3_ on tomato after 48 h of transmitting the virus; (2) comparison of the expression of the gene associated with the SA signaling pathway of tomato and the expression of the gene associated with the Toll-like signaling pathway of viruliferous whiteflies; and (3) the community composition and abundance of the microbiota (bacteria and fungi) on the surface and inside of the body of viruliferous whiteflies.

## Results

### The Changes in Whitefly Transmission Efficiency

**Figure 1:**
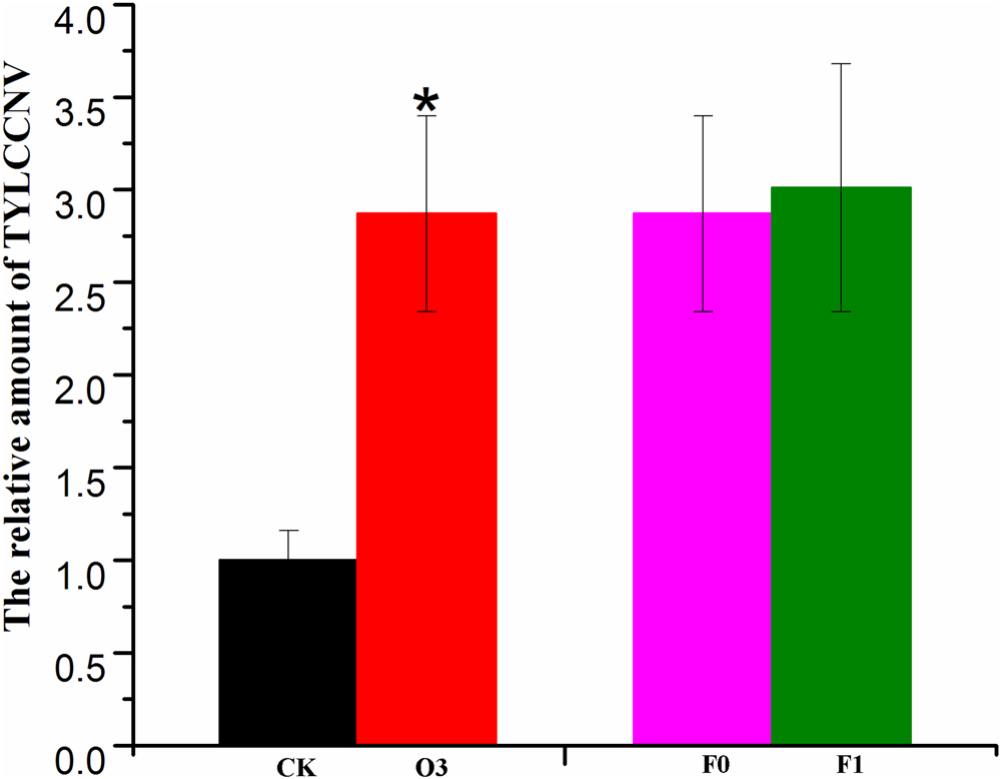
The result of the TYLCCNV transmission rates by Q biotype whiteflies from different O3 concentration and different fumigated time. Black bar is for control O3, red bar is for high O3, pink bar is for F0 generation, and green bar is for F1 generation. *P ≤ 0.05; **P ≤ 0.01; ***P ≤ 0.001.

The transmission efficiency of *TYLCCNV* by whitefly was significantly enhanced by hO3, as indicated by the significant increase up to 2.87 times, (F1,104 = 6.872, P = 0.011) of the relative amount of virus in tomato leaves which were exposed to 20 pairs of viruliferous whiteflies(Fig. 1). However, more than a generation of fumigation exposure for whitefly (*F*_1,104_ = 1.37, *P* = 0.279) and the interaction (*F*_1,104_ = 2.416, *P* = 0.100) between these two factors did not significantly affect the transmission efficiency of *TYLCCNV*.

### Relative Gene Expression of Tomato SA Resistance

**Figure 2:**
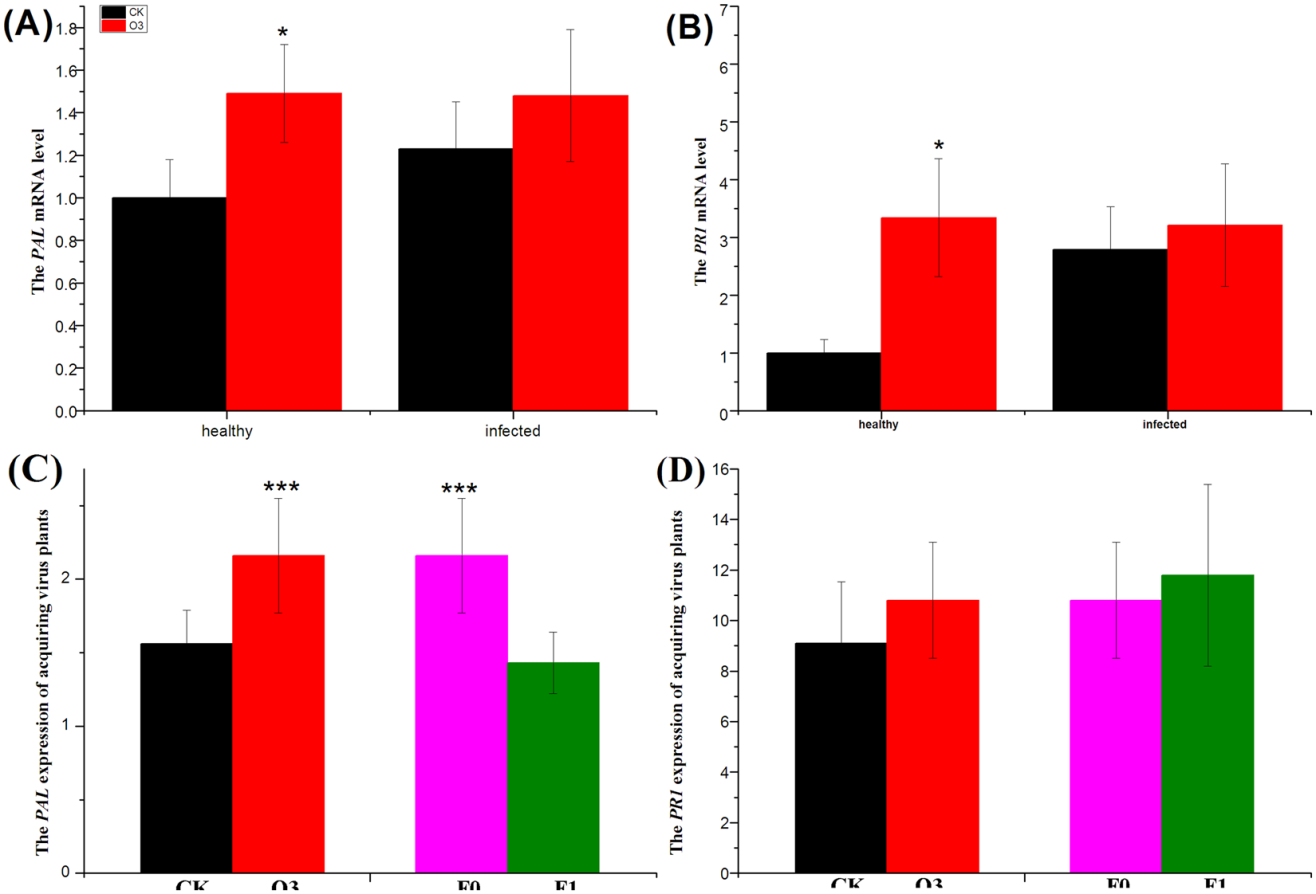
The relative expression level of SA resistance genes. (**A**) is the PAL mRNA level from healthy tomatoes and infected tomatoes by injecting TYLCCNV under high O3 and control O3; (**B**) is the PR1 mRNA level from healthy tomatoes and infected tomatoes by injecting TYLCCNV under high O3 and control O_3_; (**C**) is the PAL mRNA level from acquiring TYLCCNV by whitefly; (**D**) is the PR1 mRNA level from acquiring TYLCCNV by whitefly. Black bar is for control O_3_, and red bar is for high O_3_, pink bar is for F0 generation, green bar is for F1 generation. *P ≤ 0.05; **P ≤ 0.01; ***P ≤ 0.001.

The tomato SA resistance, as indicated by the relative amount of *PAL* and *PR*_1_ in tomato leaves, were significantly affected by high O_3_ and viruliferous whiteflies, but was not affected by TYLCCNV alone. In fact, high O_3_ significantly increased the *PAL* expression level (49.32%) of healthy tomatoes (*F*_1,32_ = 7.943, *P* = 0.0340), and high O_3_ significantly increased the *PR*_1_ expression level of healthy tomatoes up to 6.43 times (*F*_1,32_ = 13.073, *P = *0.013). High O_3_ enhanced the *PAL* expression level (8.91%) of infected tomatoes by injection (*F*_1,32_ = 0.524, *P* = 0.871), and high O_3_ enhanced the *PR*1 expression leve l (27.93%) of infected tomatoes by injection (*F*_1,32_ = 1.388, *P* = 0.267) (Fig. 2A and B). The acquiring tomato SA resistance from viruliferous whiteflies, as indicated by the relative amount of *PAL* and *PR*_1_ in tomato leaves, *PAL* was significantly affected by O_3_ concentration (*F*_1,104_ = 14.412, *P* = 0.001), more than a generation of fumigation exposure for whitefly (*F*_1,104_ = 19.115, *P* < 0.001), and the interaction between the two factors (*F*_1,104_ = 14.024, *P* < 0.001). O_3_ concentration significantly increased the *PAL* expression level up to 1.38 times, but more than a generation of fumigation exposure of high O_3_ significantly decreased the *PAL* expression level to 33.71% (Fig. 2C). The relative amount of *PR*_1_ in acquiring virus tomato leaves was high but was not affected by any one factor of O_3_ concentration (*F*_1, 104_ = 0.617, *P = *0.442), more than a generation of fumigation exposure of whitefly (*F*_1,104_ = 0.287, *P = *0.754), and the interaction (*F*_1, 104_ = 1.696, *P* = 0.211) between these two factors (Fig. 2D).

**Figure 3:**
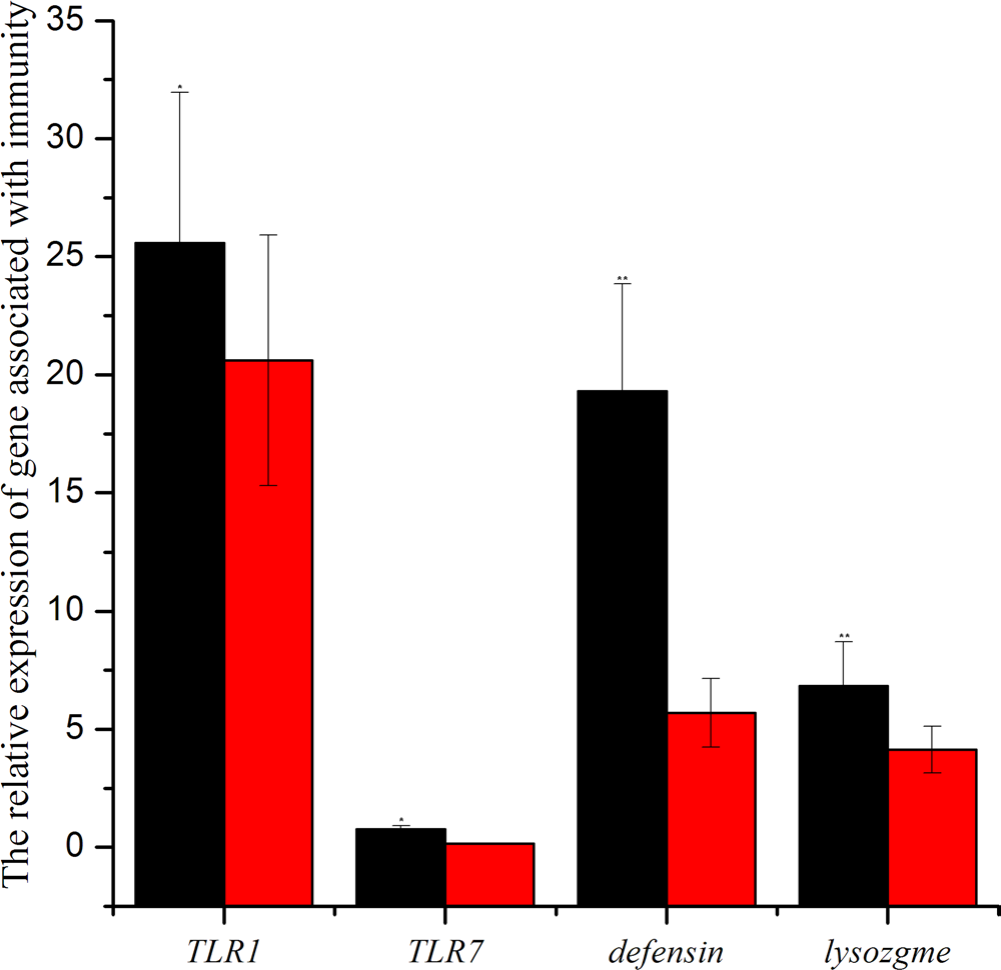
The relative expression level of four whitefly genes associated with immunity. TLR1, toll-like receptors1 gene; TLR7, toll-like receptors7 gene; Lysozgme, Lysozgme gene; defensin, antimicrobial peptides gene. *P ≤ 0.05; **P ≤ 0.01; ***P ≤ 0.001.

Relative Gene Expression Associated with the Immunity of Viruliferous Whiteflies We found that high O_3_ significantly affected the expression of genes involved in immunity, as indicated by the relative amount of *TRL*_*1*_, *TRL*_7_, *defensin* and *lysozyme* in viruliferous whiteflies. High O_3_ significantly decreased the expression of *TRL*_1_ up to 25% (*F*_1,32_ = 7.546, *P* = 0.035), the expression of *TRL*_7_ up to 21% (*F*_1,32_ = 7.396, *P* = 0.026), the expression of *defensin* up to 65.5% (*F*_1,32_ = 13.876, *P* = 0.0019), and the expression of *lysozyme* up to 58.5% (*F*_1,32_ = 9.636, *P* = 0.01) (Fig. 3). More than a generation of fumigation exposure of whitefly had similar effects with O_3_ concentration, but the data is not shown.

### Significant Changes in the Abundances of the Bacterial and Fungal Communities

**Figure 4:**
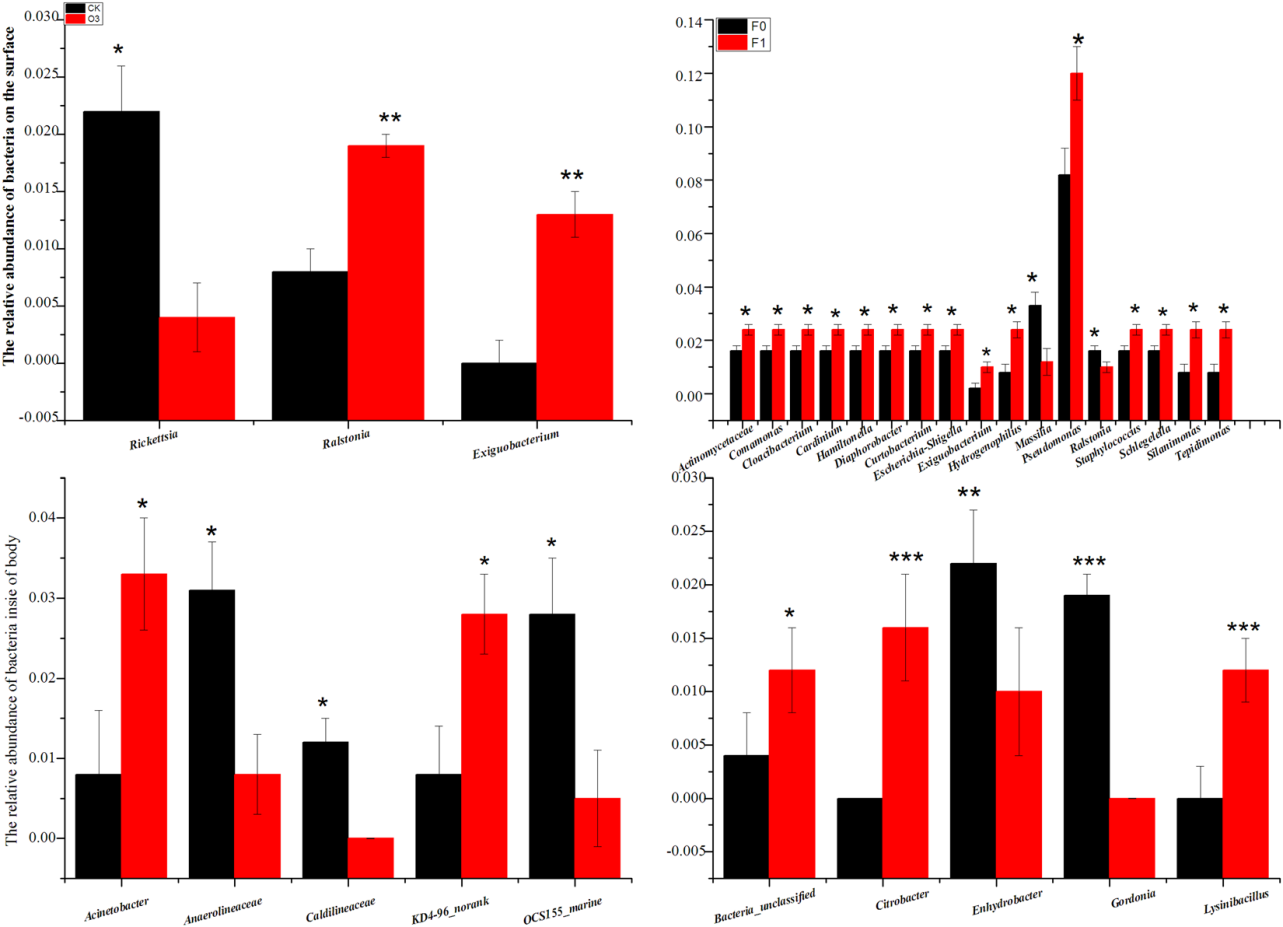
Comparing the differences of relative abundance of the bacteria. (**A**) is bacteria on the surface of whitefly from different O3 concentration, black bar is for control O3, and red bar is for high O3. (**B**) is bacteria on the surface of whitefly from different fumigated time, Black bar is bacteria from F0 generation whitefly that was for fumigated 48 h, and red bar is bacteria from F1 generation whitefly that was fumigated 48 h + 20d. (**C**) is bacteria inside the whitefly from different O3 concentration, black bar is for control O3, and red bar is for high O3. (**D**) is bacteria inside the whitefly from different fumigated time, black bar is from F0 generation whitefly that was for fumigated 48 h, and red bar is from F1 generation whitefly that was fumigated 48 h + 20 d. *P ≤ 0.05; **P ≤ 0.01; ***P ≤ 0.001.

O_3_ concentration significantly affected the mean abundance of three bacteria on the surface of whitefly. High O_3_ significantly decreased the mean abundance of *Rickettsia*, increased the mean abundance of *Ralstonia*, and newly added *Exiguobacterium* (Fig. 4A). More than a generation of fumigation exposure of whitefly significantly affected seventeen bacteria on the surface. More than a generation of fumigation exposure of whitefly significantly decreased the mean abundance of *Massilia* and *Ralstonia*. In contrast, more than a generation of fumigation exposure of whitefly significantly enhanced the mean abundance of fifteen bacteria from 1.5–5times (Fig. 4B). The interaction between the O_3_ concentration and more than a generation of fumigation exposure for whitefly significantly affected three bacteria on the surface of whitefly. They were *Ralstonia*, *Enterococcus* and *Exiguobacterium*. The O_3_ concentration significantly affected the mean abundance of five bacteria in the body of whitefly. The O_3_ concentration significantly decreased the mean abundance of three bacteria that were *Anaerolineaceae* and *OCS*1*55_marine_group*,and wiped out *Caldilineaceae*. At the same time, the O_3_ concentration significantly enhanced the mean abundance of *Acinetobacter* and *KD4–96_norank* (Fig. 4C). More than a generation of fumigation exposure of whitefly significantly affected five bacteria inside of body. More than a generation of fumigation exposure of whitefly significantly decreased the mean abundance of *Enhydrobacter* and wiped out *Gordonia*. At the same time, More than a generation of fumigation exposure of whitefly significantly enhanced the mean abundance of *Bacteria_unclassified* and new added two bacteria that was *Citrobacter* and *Lysinibacillus* (Fig. 4D). The interaction between the O_3_ concentration and more than a generation of fumigation exposure of viruliferous whitefly significantly affected four bacteria in the body of whitefly. They were *Bacteria _unclassified*, *Citrobacter*, *Enhydrobacter* and *Lysinibacillus*. The significant effects of O3 on the abundance of bacteria were shown in Table 1.

**Table 1:** ANOVA results (P-values) for the effects of O3 level, fumigation time and their interactions on bacteria.

species	location	value	Treatment_(df)_		
			O_3(1,32)_	fumigation time_(1,32)_	O_3_ × fumigation time
*Rickettsia*	on the surface	*F*	10.86		
		*P*	0.017		
*Ralstonia*	on the surface	*F*	25.938		
		*P*	0.002		
*Exiguobacterium*	on the surface	*F*	17.576		
		*P*	0.005		
*Massilia*	on the surface	*F*		7.756	
		*P*		0.032	
*Ralstonia*	on the surface	*F*		8.171	
		*P*		0.029	
*Actinomycetaceae*	on the surface	*F*		7.118	
		*P*		0.037	
*Comamonas*	on the surface	*F*		7.118	
		*P*		0.037	
*Cloacibacterium*	on the surface	*F*		7.118	
		*P*		0.037	
*Candidatus Cardinium*	on the surface	*F*		7.118	
		*P*		0.037	
*Candidatus Hamiltonella*	on the surface	*F*		7.118	
		*P*		0.037	
*Diaphorobacter*	on the surface	*F*		7.118	
		*P*		0.037	
*Curtobacterium*	on the surface	*F*		7.118	
		*P*		0.037	
*Escherichia -Shigella*	on the surface	*F*		7.118	
		*P*		0.037	
*Pseudomonas*	on the surface	*F*		7.118	
		*P*		0.037	
*Staphylococcus*	on the surface	*F*		7.118	
		*P*		0.037	
*Schlegelella*	on the surface	*F*		7.118	
		*P*		0.037	
*Tepidimonas*	on the surface	*F*		7.118	
		*P*		0.037	
*Hydrogenophilus*	on the surface	*F*		12.386	
		*P*		0.013	
*Silanimonas*	on the surface	*F*		12.386	
		*P*		0.013	
*Exiguobacterium*	on the surface	*F*		7.034	
		*P*		0.038	
*Enterococcus*,*0*.*48*	on the surface	*F*			20.71
		*P*			0.004
*Exiguobacterium*	on the surface	*F*			7.034
		*P*			0.038
*Ralstonia*,*0*.*53*	on the surface	*F*			21.109
		*P*			0.004
*Anaerolineaceae*	in the body	*F*	8.172		
		*P*	0.021		
*OCS155_marine*	in the body	*F*	5.78		
		*P*	0.043		
*Caldilineaceae*	in the body	*F*	8.83		
		*P*	0.018		
*Acinetobacter*	in the body	*F*	5.624		
		*P*	0.045		
*KD4-96_norank*	in the body	*F*	7.11		
		*P*	0.029		
*Enhydrobacter*	in the body	*F*		20.91	
		*P*		0.002	
*Gordonia*	in the body	*F*		45.043	
		*P*		0.0001	
*Bacteria_unclassified*	in the body	*F*		7.396	
		*P*		0.026	
*Lysinibacillus*	in the body	*F*		364.608	
		*P*		0.0001	
*Citrobacter*	in the body	*F*		5.473	
		*P*		0.047	
*Bacteria_unclassified*	in the body	*F*			7.396
		*P*			0.026
*Lysinibacillus*	in the body	*F*			364.608
		*P*			0.0001
*Citrobacter*	in the body	*F*			5.473
		*P*			0.047
*Enhydrobacter*	in the body	*F*			20.891
		*P*			0.002

**Figure 5:**
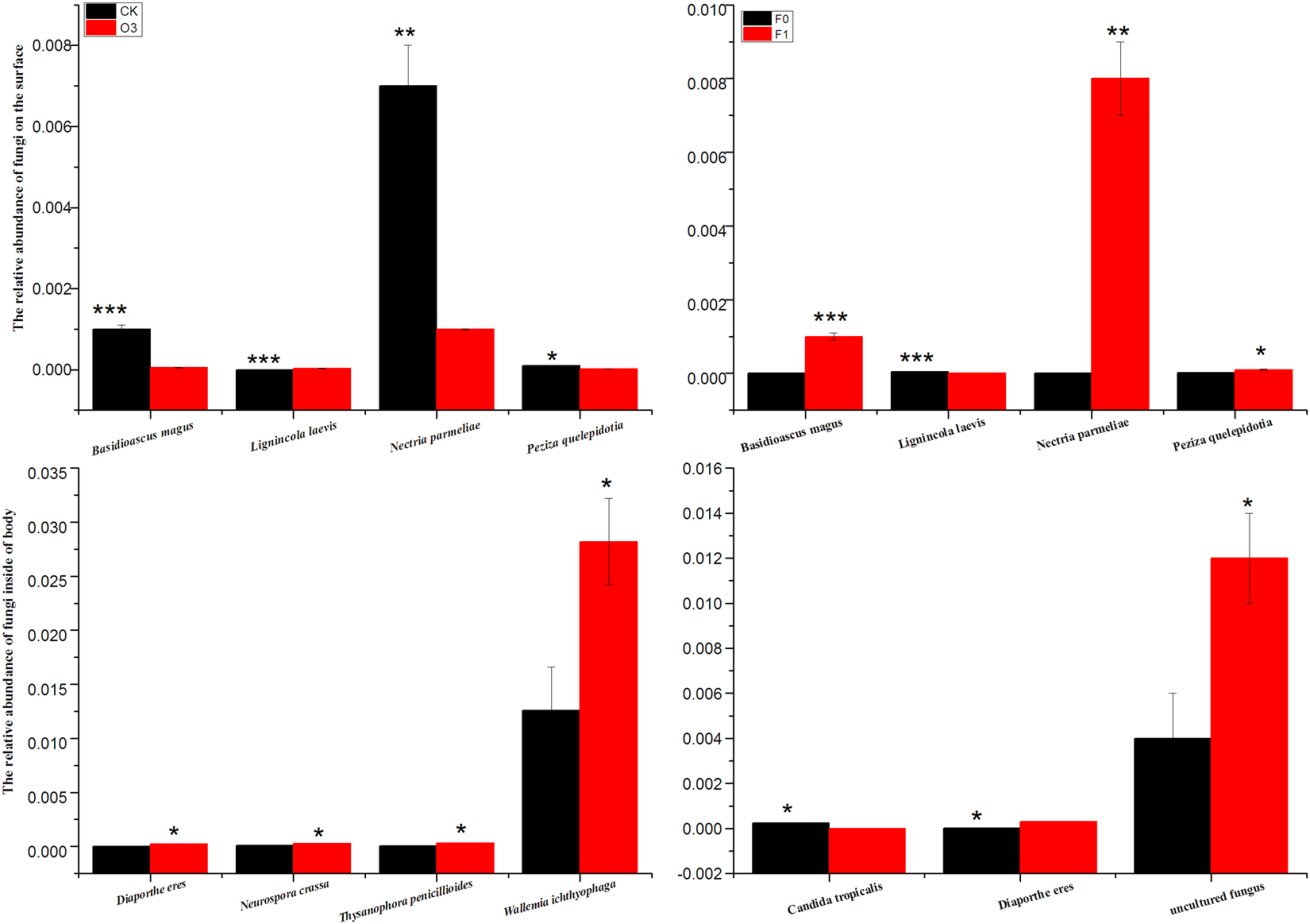
Comparing the differences of relative abundance of the fungi on the base of the mean value. (**A**) is fungi on the surface of whitefly from different O3 concentration, black bar is for control O3, and red bar is for high O3. (**B**) is fungi on the surface of whitefly from different fumigated time, black bar is fungi from F0 generation whitefly that was for fumigated 48 h, and red bar is fungi from F1 generation whitefly that was fumigated 48 h + 20d. (**C**) is fungi inside the whitefly from different O3 concentration, black bar is for control O3, and red bar is for high O3. (**D**) is fungi inside the whitefly from different fumigated time, black bar is from F0 generation whitefly that was for fumigated 48 h, and red bar is from F1 generation whitefly that was fumigated 48 h + 20d. *P ≤ 0.05; **P ≤ 0.01; ***P ≤ 0.001.

O_3_ concentration, more than a generation of fumigation exposure of viruliferous whitefly, and the interaction between the two factors significantly affected the same four fungi on the surface of whitefly that were *Basidioascus magus*, *Lignincola laevis*, *Nectria parmeliae* and *Peziza quelepidotia*; the significance level was similar (Fig. 5A and B). O_3_ concentration significantly enhanced the mean abundance of four fungi in the body of whitefly from 2.24 to 19.2 times. They were *Diaporthe eres*, *Neurospora crassa*, *Thysanophora penicillioides* and *Wallemia ichthyophaga* (Fig. 5C). More than a generation of fumigation exposure wiped out *Candida tropicalis* and added *Diaporthe eres* (Fig. 5D). The interaction between the two factors significantly affected the mean abundance of three fungi in the body of whitefly. They were *Basidioascus magus*, *Diaporthe eres* and *Neurospora crassa*. The significant effects of O3 on the abundance of fungi were shown in Table 2.

**Table 2:** ANOVA results (P-values) for the effects of O3 level, fumigation time and their interactions on fungi.

species	location	value	Treatment_(df)_		
			O_3(1,32)_	fumigation time_(1,32)_	O_3_ × fumigation time
*Basidioascus magus*	on the surface	*F*	694.58		
		*P*	0.001		
*Lignincola laevis*	on the surface	*F*	42.632		
		*P*	0.001		
*Nectria parmeliae*	on the surface	*F*	20.79		
		*P*	0.004		
*Peziza quelepidotia*	on the surface	*F*	8.172		
		*P*	0.021		
*Basidioascus magus*	on the surface	*F*		805.456	
		*P*		0.001	
*Lignincola laevis*	on the surface	*F*		38.806	
		*P*		0.001	
*Nectria parmeliae*	on the surface	*F*		29.442	
		*P*		0.002	
*Peziza quelepidotia*	on the surface	*F*		8.172	
		*P*		0.021	
*Basidioascus magus*	on the surface	*F*			677.43
		*P*			0.001
*Lignincola laevis*	on the surface	*F*			57.935
		*P*			0.001
*Nectria parmeliae*	on the surface	*F*			15.339
		*P*			0.008
*Peziza quelepidotia*	on the surface	*F*			20.445
		*P*			0.004
*Diaporthe eres*	in the body	*F*	13.685		
		*P*	0.014		
*Neurospora crassa*	in the body	*F*	8.393		
		*P*	0.034		
*Thysanophora penicillioides*	in the body	*F*	15.555		
		*P*	0.011		
*Wallemia ichthyophaga*	in the body	*F*	8.51		
		*P*	0.033		
*Candida tropicalis*	in the body	*F*		8.482	
		*P*		0.033	
*Diaporthe eres*	in the body	*F*		13.685	
		*P*		0.014	
*Basidioascus magus*	in the body	*F*			10.207
		*P*			0.024
*Diaporthe eres*	in the body	*F*			16.166
		*P*			0.01
*Neurospora crassa*	in the body	*F*			22.177
		*P*			0.005

## Discussion

It is well known that host plant resistance can affect the transmission rates of viruses by vector insects. In addition, plant resistance is affected by various biotic and abiotic factors including atmospheric changes, herbivore insects and microorganisms. Our previous studies proved the elevated CO_2_ (750 ppb) and O_3_ (80 ppb) enhanced the resistance of tomato plants fumigated for 20–40 days by means of jasmonic acid defenses (JA) or salicylic acid defenses (SA) signaling pathways^7,26^. Zarate *et al*.^34^ found that whitefly-induced SA defenses suppressed the JA defenses of the host plant. Viruses can also affect host plant SA resistance and the virus transmission rate^35,36^. In the study, high O_3_ (≥280 ± 20ppb,48 h) as the sole abiotic factor significantly affect the resistance of healthy tomato plants, the results was same with early studies^7,26^. High O_3_ as an abiotic factor and whitefly and TYLCCNV as biotic factors affected the SA resistance of acquiring virus tomato plants, significantly up-regulating the *PAL* mRNA level of tomato plants, and the results were similar with a previous study that reported that 200 ppb O_3_ significantly enhanced the SA level of tobacco and *Arabidopsis* ^37,38^. However, O_3_ did not have a significant effect on the down-stream gene of SA (*PR*_1_), which might be due to viruliferous whiteflies as the community of insects and the virus could overlap the effects on the mRNA of *PR*_1_, making the mRNA of *PR*_1_ of acquiring virus tomato plants reach big value under control O_3_. The results indicated that high O_3_ had a less direct effect on the SA resistance of receptor plants than viruliferous whiteflies did. Viruliferous whiteflies with TYLCCNV should have same effects on the SA resistance of receptor plants under the same O_3_ concentration. Why did the F_0_ generation (O_3_ fumigation for 48 h) of viruliferous whitefly significantly up-regulate the *PAL* mRNA level of the receptor plants, but the F_1_ generation (O_3_ fumigation for 20d + 48 h) of viruliferous whitefly significantly down-regulate the *PAL* mRNA level of the receptor plants under same O_3_ concentration? We thought that high O_3_ altered the SA resistance of the receptor plants by regulating the composition and abundance of the microbia associated with whiteflies. In fact, some insect symbiotic microorganisms may have influenced the SA defense of the receptor plants^12,18^. In the study, the O_3_ concentration significantly increased the abundance of *Ralstonia*, and prolonged O_3_ fumigation exposure time significantly decreased the abundance of *Ralstonia* on the surface of the whitefly. Similarly, the O_3_ concentration and the O_3_ fumigation exposure time had contrary effects on the abundance of four fungi (*Basidioascus magus*, *Lignincola laevis*, *Nectria parmeliae* and *Peziza quelepidotia*) on the surface of whitefly. Because the change trends of the abundances of *Ralstonia*, *Basidioascus magus*, *Lignincola laevis*, *Nectria parmeliae* and *Peziza quelepidotia* agreed with that of the *PAL* mRNA levels between O_3_ concentration and O_3_ fumigation exposure time, we speculated that *Ralstonia*, *Basidioascus magus*, *Lignincola laevis*, *Nectria parmeliae* and *Peziza quelepidotia* might play major roles in ozone-regulated receptor plant resistance. Studies showed that *Ralstonia* could degrade phenol and *Nectria parmeliae* could produce cell wall-degrading enzymes^39,40^. Such research might explain why O_3_ fumigation exposure time and O_3_ concentration had different effects on the SA defense of acquiring virus tomatoes. However, it is necessary for us to further confirm the functions of *Basidioascus magus*, *Lignincola laevis*, *Nectria parmeliae* and *Peziza quelepidotia* on SA resistance in plants in the future.

Sade *et al*.^8^ found SA was involved in tomato resistance to Tomato Yellow Leaf Curl Virus (TYLCV), and the TYLCV transmission rate by whiteflies was negatively correlated with the SA resistance of the receptor plants. That is, the higher the resistance, the lower the transmission rate of TYLCV by whiteflies^41^. However, in the study, the resistance of tomato plants increased under high O_3_, and at the same time, the transmission rate of TYLCV by whiteflies increased under high O_3_. What brought about the contradictory results? The transmission rate of TYLCV was not only affected by the resistance of the receptor plants but was also affected by symbiotic microorganisms associated with the vector insects. Some insect symbiotic microorganisms may influence the transmission of a virus by regulating the SA or JA defenses of the receptor plants^12,18^; some insect symbiotic microorganisms may work via the secretion of a capsid protein to help transmit the plant virus^20^. Studies have shown that *Ralstonia* and *Diaphorobacter* could degrade phenol and decrease plant SA resistance^39,42^, *Basidioascus* could resist environmental stress^43^, *Lignincola laves* and *Wallemia ichthyophaga* can resist hypersaline^44^, *Nectria parmeliae* could produce cell wall-degrading enzymes and decrease the plant resistance^45^, *Diaporthe eres* can degrade 3-nitrotoluene^46^, and *Thysanophora penicillioides* can affect whitefly immunity^10^. The changes in composition or abundance of these bacteria and fungi could affect plant and insect resistance to stress, which could change the transmission virus rate by the vector insects. In the study, high O_3_ significantly increased the abundance of *Ralstonia*, *Diaphorobacter* and *Nectria parmeliae* on the surface of whitefly, while decreasing the acquired TYLCCNV tomato resistance and increasing the TYLCCNV transmission rate by whitefly. High O_3_ significantly increased the abundance of *Basidioascus*, *Wallemia ichthyophaga*, *Diaporthe eres* and *Thysanophora penicillioides*, thus enhancing whitefly fittness under high O_3_ and increasing the TYLCCNV transmission rate by whitefly.

Moreover, high O_3_ fumigation for 48 h only significantly decreased the abundance of *Rickettsia* on the surface of whitefly and significantly enhanced the abundance of *Hamiltonella* in the body of whitefly. The results proved the points of view that *Rickettsia* and *Hamiltonella* affected the TYLCV transmission rate^20,21,23,25^. In the study, *Rickettsia* showed a positive with the immunity of whitefly, the result was agreed with our previously study^10^. Early study showed *Rickettsia* was a positive with TYLCV transmission rate^21^. However, in the study, *Rickettsia* showed a negative transmission TYLCV rate, that is, high O_3_ significantly decreased the abundance of *Rickettsia* and significantly increased the TYLCV transmission rate. We thought that high O_3_ decreased the immunity of whitefly, as a result, indirectly promoted the TYLCV transmission. And same symbiotic bacteria had different biological effect in different hosts. In the study, high O_3_ enhanced the abundance of catalase positive bacteria; for example, *Ralstonia* and *Exiguobacterium* were enhanced on the surface of whitefly, and *Acinetobacter* and *KD4–96_norank* were enhanced in the body of whitefly. In addition, the prolonged O_3_ fumigation exposure time enhanced the abundance of more catalase positive bacteria. For instance, the prolonged O_3_ fumigation exposure time significantly enhanced the abundance of nine catalase positive bacteria on the surface and three catalase positive bacteria in the body of whitefly. They were *Staphylococcus*, *Pseudomonas*, *Micrococcus*, *Escherichia – Shigella*, *Curtobacterium*, *Cloacibacterium*, *Actinomycetaceae*, *Ralstonia*, *Exiguobacterium*, *Citrobacter*, *Enhydrobacter*, *and Lysinibacillus*. The results demonstrated that O_3_ made organisms generate a lot of hydrogen peroxide. This might partly explain why O_3_ fumigation exposure time and O_3_ concentration had different effects on the TYLCCNV transmission rate. In the future, we could validate the special function of the TYLCCNV transmission rate of bacteria and fungi that demonstrated significant differences under high O_3_ and long O_3_ fumigation exposure time.

## Materials and Methods

### O_3_ Treatment

In the hO_3_ treatment, O_3_ was generated from ambient air with an O_3_ generator (3S-A10, Beijing Ligong University, Beijing, China) and then transported into an artificial climate chamber at 25 ± 1 °C, 70% RH and a 14:10 L:D photoperiod (PRX-450C, Ningbo, China). The O_3_ concentrations were monitored in real time (Shenzhen Yiyuntian Electronic CO. LTD). In the treatment, O_3_ was ventilated from 9:00 a.m. to 5:00 p.m, and an artificial climate chamber at 25 ± 1 °C, 70% RH and a 14:10 L: D photoperiod (PRX-450C, Ningbo, China) without an O_3_ generator was used as a control. In the study, we used four chambers for high O3 treatment and four chambers for control treatment, three high O3 arrangements and three control arrangements were used to cultivate plant, the other two arrangements were used to train viruliferous whiteflies.

### Healthy Plants, Whiteflies and TYLCCNV bacterial liquid

Tomato seeds were sown in 10 cm diameter plastic pots with commercial peat soil, and the pots was incubated in a screened cage (60 × 40 × 40 cm, 100 mesh) in a greenhouse. The plants were watered every two days. To conduct detoxification treatment, Q-biotype *B*. *tabaci* fed on an 80 cm-high cotton plant in a screened cage (150 × 100 × 100 cm, 100 mesh) in a greenhouse. The Q-biotype *B*. *tabaci* and TYLCCNV bacterial liquid were obtained from the Institute of Vegetables and Flowers of the Chinese Academy of Agricultural Sciences (CAAS) on April 22, 2015, and they were identified by PCR. TYLCCNV bacterial liquid means Agrobacterium tumefaciens carrying TYLCCNV and betasatellite DNAβ. Agroinfiltration was performed with an overnight culture as described previously^47,48^.

### High O_3_ Fumigation for Infected and Healthy Tomato Plants

A week after injecting TYLCCNV bacterial liquid, eighteen infected tomato plants and eighteen healthy tomato plants confirmed by PCR were transferred to three high O_3_ (280 ± 20 ppb, hO_3_) arrangements and three control O_3_ (50 ± 10 ppb) arrangements (six infected tomato plants and six healthy tomato plants in each arrangement), respectively. After 48 h, 1–2 new leaves were collected from the infected and healthy tomato plants. To determine whether the SA resistance of the tomato plants came from high O_3_ or from TYLCCNV alone, we determined the mRNA levels of the genes of phenylalanine ammonia lyase (*PAL*) and pathogenesis related protein_1_ (*PR*_1_) from the infected or healthy tomatoes leaves using real-time quantitative RT-PCR (qRT-PCR) as described in the next section. The experiment was repeated three times.

### Viruliferous Plants and Viruliferous Insects

First, 200 μl bacterial liquid with TYLCCNV DNA was injected to 24 healthy tomato plants at the three-true-leaf stage and were incubated in a screened cage (60 × 40 × 40 cm, 100 mesh) in a greenhouse. Infected plants were confirmed by the appearance of typical leaf curl symptoms and PCR. Approximately 1000 pairs of newly eclosed whitefly adults were released onto 6 viruliferous plants 20 days after injection. Then, 3 viruliferous plants with whiteflies were transferred to a high O_3_ (280 ± 20 ppb, hO_3_) arrangement, and 3 viruliferous plants with whiteflies were transferred to a control O_3_ (50 ± 10 ppb, control O_3_) arrangement.

### Transmission of TYLCCNV by Whiteflies

Twenty pairs of randomly selected viruliferous Q-biotype *B*. *tabaci* were transferred to healthy tomato leaves covered with bags (40 × 50 cm, 100 mesh). Six healthy tomato plants (three bags/plant) with viruliferous Q-biotype *B*. *tabaci* were transferred to the hO_3_ and control O_3_ arrangements. After 48 h, the bag with acquiring virus tomato leaves and the transmitting virus whiteflies from each plant was collected, and the experiment was repeated three times. All live whiteflies from a plant (3 bags) were used to extract the total RNA of the whitefly and the DNA of the microbial community on the surface and in the body of the whitefly, as described in the next section. The RNA of the whitefly was used to assess the relative gene (Toll-like receptor 1 (*TLR*_*1*_), Toll-like receptor 7 (*TLR*_7_), *defensin* and *lysozyme*) expressions in the toll pathway. At the same time, the RNA and DNA in the leaves from a bag were extracted, and the relative gene expression of SA resistance and the TYLCCNV DNA by qRT-PCR and q-PCR were assessed as described in the following section.

### Relative Gene Expression Associated with SA Resistance in Tomato and the Toll Pathway of Whitefly and TVLCCNV DNA by qRT-PCR and q-PCR

To explore whether hO_3_ affected TYLCCNV transmission by whiteflies, we compared the TYLCCNV DNA of the acquiring virus tomato leaves from hO_3_ and the control O_3_ using real-time quantitative PCR (q-PCR). DNA was extracted from the leaves with a plant genomic DNA kit (Tiangen Biotech, Beijing, Co., Ltd). To explore whether the SA resistance of tomatoes result from hO_3_ or TYLCCNV alone, we determined the mRNA levels of the *PAL* and *PR*_1_ genes from healthy and infected tomatoes from hO_3_ and the control O_3_ using real-time quantitative RT-PCR (qRT-PCR). Moreover, in order to determine whether the resistance and acquiring virus effects of the receptor plants were mainly affected by the direct effect of hO_3_ or the indirect effect of hO_3_ via affecting microbes associated with whitefly, we compared the TYLCCNV DNA and the mRNA levels of the SA signaling pathway of acquiring virus tomatoes transmitted by viruliferous Q-biotype *B*. *tabaci* that were fumigated for 48 h (F_0_ generation) and were fumigated for 48 h + 20 days (F_1_ generation). To explore whether changes in gene expression altered viruliferous whitefly immunity under hO3, we determined the mRNA levels of the toll-like receptor 1 (*TLR*1) and the toll-like receptor 7 genes (*TLR*7), *defensin* and lysozyme (*LYS*) by real-time quantitative RT-PCR (qRT-PCR). The total RNA of the live viruliferous whitefly samples and the total RNA of acquiring virus tomato leaves from the high O_3_ treatment and the control O_3_ were extracted by TRIzol (Invitrogen) according to the manufacturer’s protocols. The RT-qPCR reaction conditions were previously described^10^ by Hong *et al*.^10^. Twenty-seven biological replicates with three technical replicates from high O_3_ and control O_3_ were performed in a generation. The relative quantification was performed using the Livak method (2^−△△C^_T_), and the values obtained were normalized to the housekeeping genes.

### The Composition and Abundance of the Microbial Communities

To explore whether changes in the microbial communities altered TYLCCNV transmission by whitefly under hO_3_, we determined the composition and abundance of the bacterial and fungal communities on the surface and the inside of the body of the whiteflies from the hO_3_ treatment (280 ± 20 ppb) and the control (50 ± 10 ppb) using 16S and 18S sequencing, which were conducted according to previously described^10^. Because the total microbial DNA on the surface of whitefly was very low, about 60% live whiteflies from acquiring virus tomatoes were used as a microbial sample (bacteria and fungus). In a generation, nine microbial samples from the treatment and control were used to analyze the composition and abundance of the bacterial and fungal communities on the surface and inside the body of the whiteflies from the hO_3_ treatment and the control O_3_. In all, thirty-six microbial samples were analyzed.

### Statistical Analyses

Multivariate analysis under the general linear model was used to analyze the hO_3_ effects on the content of TYLCCNV, gene expression, and the relative abundance of the microbial community; the level of significance was set at P < 0.05. The fitting agenda was based on the mean of the corresponding element. The statistical analyses were conducted using SPSS 19.0 (IBM, USA).
